# A nomogram for prediction of stage III/IV gastric cancer outcome after surgery: A multicenter population‐based study

**DOI:** 10.1002/cam4.3215

**Published:** 2020-06-15

**Authors:** Zhiying Gao, Jing Ni, Hui Ding, Caiwang Yan, Chuanli Ren, Gang Li, Feng Pan, Guangfu Jin

**Affiliations:** ^1^ Department of Epidemiology and Biostatistics Center for Global Health School of Public Health Nanjing Medical University Nanjing China; ^2^ Department of Gastroenterology The Affiliated Huaian No.1 People’s Hospital of Nanjing Medical University Huai'an China; ^3^ Jiangsu Key Lab of Cancer Biomarkers, Prevention and Treatment Collaborative Innovation Center for Cancer Medicine Nanjing Medical University Nanjing China; ^4^ Clinical Medical Testing Laboratory Northern Jiangsu People's Hospital and Clinical Medical College of Yangzhou University Yangzhou China; ^5^ Department of General Surgery Jiangsu Cancer Hospital Jiangsu Institute of Cancer Research The Affiliated Cancer Hospital of Nanjing Medical University Nanjing China

**Keywords:** nomogram, prognosis, SEER database, stomach neoplasm

## Abstract

Most patients with gastric cancer (GC) are first diagnosed at stage III‐IV and surgery resection remains the primary therapeutic modality for these patients. However, clinical staging used for prediction of those patients provides limited information. We collected clinicopathological data and disease‐progression information from 508 patients with stage III‐IV GC at three Chinese hospitals and 1298 patients from the Surveillance, Epidemiology, and End Results database. Based on the stepwise multivariate regression model, we constructed a novel nomogram to predict overall survival (OS). The performance of discrimination for this model was measured using Harrell's concordance index (C‐index) and receiver‐operating characteristic curve (ROC), and was validated using calibration plots. Multivariate Cox regression analyses showed that tumor size, age at diagnosis, N stage, tumor grade, and distant metastases were outstanding independent prognostic factors of stage III‐IV GC. We developed a nomogram based on these five prognostic predictors. In the training set, the C‐index of the nomogram was 0.645 (95% CI: 0.611‐0.679), which was higher than that of the American Joint Committee on Cancer TNM system alone (sixth TNM: 0.544; seventh TNM: 0.575; eighth TNM: 0.568). Similar results were observed in validation cohort. Moreover, calibration blots demonstrated good consistency between the actual and predicted OS probabilities. According to the nomogram, GC individuals could be classified into three groups (low‐, middle‐, and high‐risk) (*P *< .001). Our nomogram complements the current staging system for prediction of individual prognosis with stage III‐IV GC, and may be helpful for making individualized treatment decisions.

## Graphical abstract

We developed a nomogram for outcome of stage III/IV gastric cancer after surgery based on patients at three Chinese hospitals and SEER database for external validation. This nomogram can complement the current staging system which may benefit individualized treatment decisions.

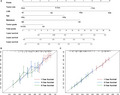

## 1 INTRODUCTION

Gastric cancer (GC) is one of the deadliest tumors around the world.^1^ Most patients with GC are first diagnosed at stage III‐IV and the outcomes in these patients are poor.^2^ Surgery appears to be a good treatment strategy for such patients.^3^ Nevertheless, there are few studies with a focus on the prediction of overall survival (OS) of stage III‐IV GC patients who underwent radical surgical treatment.^4^

The tumor‐node‐metastases (TNM) staging system is the key determinant for the prediction of survival and decisions regarding clinical treatment.^5^ However, there are significantly different prognoses among patients at the same stage, especially advanced GC. Age, gender, tumor grade, and tumor size may account for this phenomenon.^6^, ^7^, ^8^, ^9^ The gastric cancer staging system based on American Joint Committee on Cancer staging systems (AJCC) has constantly altered over the years reaching its recent eighth edition. The major change from seventh system is the separation of N3a (7‐15 positive regional lymph nodes) and N3b (>15 positive regional lymph nodes) in the eighth TNM classification.^10^ A retrospective control study showed that the eighth AJCC‐TNM system can better determine the outcomes of GC patients.^11^ Therefore, developing a tool that integrates multiple confirmed prognostic factors into a single numerical estimate of survival may be helpful for individualized treatment decisions and postoperative counseling.

Nomograms have been described as alternatives and even as new standards for the management of several cancers, including liver, colon, and breast cancer.^12^, ^13^, ^14^ A large number of clinical studies have confirmed that a nomogram integrated with multiple variables achieved better prognostic predictions than with TNM systems alone.^15^ For stage II‐III GC patients, a nomogram showed more accurate predictive ability than the TNM stage alone based on systemic prognostic score, TNM stage, and tumor location (Harrell's concordance‐index; C‐index: nomogram 0.714 vs 8th TNM 0.630; *P *< .001).^16^ Nevertheless, few studies focused on predicting OS after surgery for III‐IV GC patients, particularly in the Chinese population.

Therefore, we investigated the independent prognostic factors and constructed a nomogram with multiple variables to predict outcomes of stage III‐IV GC patients after surgery, which complements the current staging system for prediction of individual prognosis with stage III‐IV GC.

## 2 MATERIALS AND METHODS

### 2.1 Patients

**FIGURE 1:**
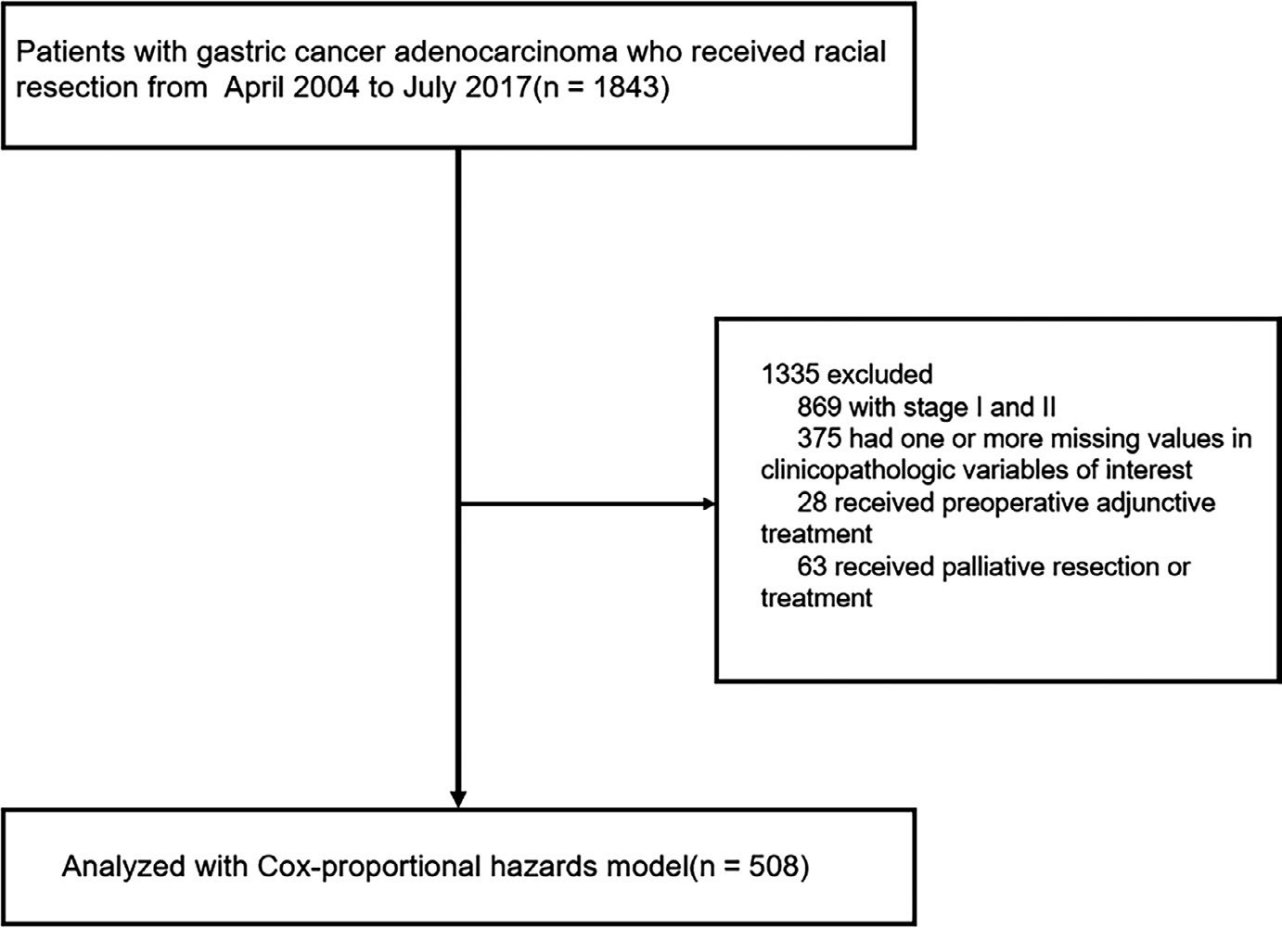
Selection process of subjects for the Cox regression model and construction of nomogram

We collected information for 1843 patients with GC who underwent complete gastric resection between April 1, 2004 and July 1, 2017, as routine surveillance population at three hospitals in China: The First Affiliated Hospital of Nanjing Medical University, The Northern Jiangsu People's Hospital and The Cancer Hospital of Nanjing medical University. Our study was approved by the Institutional Review Board of Nanjing Medical University (FWA00001501). After signing informed consent, we selected patients (older than 18 years) who met the following inclusion criteria: (1) stomach adenocarcinoma confirmed by at least two pathologists; (2) no combined malignancy; (3) definite pathological information for T stage, number of positive lymph nodes and distant metastasis; and (4) clinical stage III or IV after local or distant curative surgery. Finally, a total of 508 patients with available clinicopathological characteristics and follow‐up information were included in the training cohort (Figure 1).

Then, we selected GC patients (older than 18 years) who underwent racial resection between January 1, 2004 and December 1, 2013 from the Surveillance, Epidemiology, and End Results (SEER) database as the external validation set. The inclusion criteria were as follows: (1) patients at stage III‐IV received radical surgery; (2) gastrectomy with > 16 nodes examined; (3) adequate follow‐up; and (4) confirmed single primary GC (ICD‐O‐3 codes: 8010 − 8231 and 8255‐8576). A total of 1298 patients with newly diagnosed stage III‐IV were finally enrolled in the external validation cohort.

All the patients in our study were reclassified according to the eighth, seventh, and sixth editions of the AJCC staging system.^17^, ^18^, ^19^

### 2.2 Data collection

First, clinical data including gender, age, and years at diagnosis of patients were recorded. Second, according to their pathological reports, we recorded tumor differentiation (G1, G2, G3), the maximum diameter of the primary tumor, number of positive lymph nodes (N stage), tumor location (upper (U), middle (M), or lower (L) portion of the stomach), depth of tumor invasion (T stage), and distant metastasis (M). Third, all patients in the training cohort underwent a standard following up process after surgery to obtain accurate survival information.

We followed up each patient every 6 months through telephone contacts until death or loss to follow‐up (until July 15, 2019). Information on adjuvant treatment (radiotherapy and chemotherapy) and survival information (cause of death, time of death, and alive, dead,) were collected. Definition of the OS was the period between the last resection surgery and date of death or loss to follow‐up (until July 2019).

### 2.3 Construction of the nomogram

We classified continuous variables (age, lymph nodes) as classification variables. To stratify patients at different risks, cutoff points for age and tumor size were selected using the X‐tile program.^20^ Age was divided into two parts using the cutoff of 63 years (Figure S1A). Tumor size was also classified into three groups using cutoffs 5.0 and 7.0 cm (Figure S1B). The Kaplan‐Meier (KM) method was used to draw survival curves and estimate the median survival time (MST) and OS for each variable. Univariate analysis by log‐rank test was used to assess the significance of each variable in the training cohort. The stepwise multivariate regression model was applied using variables with *P*‐values < 0.05 to identify independent predictors. We selected the final model by a backward step‐down process with the Akaike information criterion (AIC).^21^ The nomogram is based on proportionally converting each regression coefficient in multivariate analysis to a 0‐ to 10‐ point scale. The effect of the variable with the highest β coefficient is assigned 10 points and the points are added to obtain total points, which are converted to predicted probabilities (Total points = 3.33 × LNM +4.74 × Age +6.59 × Metastasis +2.32 × Tumor grade + 4.00 × Tumor size).

### 2.4 Model performance

C‐index was used to evaluate discrimination of novel model performance. We compared the discriminative abilities of the nomogram using the sixth, seventh, and eighth TNM staging systems.^22^ Calibration plots for 1‐, 3‐, and 5‐year OS probability were drawn to compare the predicted event and actual event. Receiver operating characteristic curves (ROC) and area under curve (AUC) were used to assess the model's ability to distinguish events and nonevents. The “*Rcorrp.cen*s” package in R was used to compare the nomogram and other models. Bootstrapping (1000 resamples) was used for bias correction. Additionally, a risk classification system for advanced GC was generated by X‐tile program according to the calculated total points of each patient by using the nomogram for clinical use.

### 2.5 Statistical analysis

All the data were imported by EpiData (version 3.0.2) software. All tests were significant at *P *< .05. All statistical analyses were performed by R software (version 3.6.3) (https://www.r‐project.org/).

## 3 RESULTS

### 3.1 Patient features

In total, 1806 GC patients at stage III‐IV who received surgical resection were analyzed (508 for the training cohort and 1298 for the validation cohort). The median follow‐up time was 83.9 months (range: 13.1‐172.3 months) for the training cohort and 21.0 months (range: 4.0‐154.0 months) for the validation cohort. The median survival time of the training and validation cohort were 44.4 months and 21.0 months, respectively. The characteristics of two cohorts were listed in Table 1.

**TABLE 1:** Baseline characteristics of study patients

Characteristics	Training cohort				Validation cohort	
	Patients	Deaths	MST (months)	Patients	Deaths	MST (months)
	N = 508 (%)	N = 295 (%)		N = 1298 (%)	N = 1017 (%)	
Age (year)						
<63	232 (45.67)	116 (39.32)	58.6	542 (41.76)	375 (36.87)	28.0
≥63	276 (54.33)	179 (60.68)	37.5	756 (58.24)	642 (63.13)	18.0
Gender						
Male	385 (75.79)	228 (77.29)	42.1	831 (64.02)	642 (63.13)	22.0
Female	123 (24.21)	67 (22.71)	54.3	467 (35.98)	375 (36.87)	20.0
Location						
Upper third (C)	257 (50.59)	150 (50.85)	45.3	326 (25.12)	230 (22.62)	25.0
Middle third (M)	152 (29.92)	81 (27.46)	58.6	452 (34.82)	356 (35.00)	20.0
Lower third (A)	94 (18.50)	60 (20.34)	34.0	520 (40.06)	431 (42.38)	20.0
Missing	5 (0.98)	4 (1.36)	–	–	–	–
Tumor size (cm)						
<5	178 (35.04)	94 (31.86)	65.5	396 (30.51)	307 (30.19)	23.0
5‐7	261 (51.38)	148 (50.17)	39.6	551 (42.45)	424 (41.69)	21.0
>7	69 (13.58)	53 (17.97)	19.8	351 (27.04)	286 (28.12)	19.0
Tumor grade						
G1/G2	298 (58.66)	167 (56.61)	49.5	376 (28.97)	278 (27.34)	25.0
G3	210 (41.34)	128 (43.39)	42.1	922 (71.03)	739 (72.66)	20.0
T stage						
T1	–	–	–	7 (0.54)	6 (0.59)	10.0
T2	–	–	–	49 (3.78)	33 (3.24)	27.0
T3	–	–	–	571 (43.99)	439 (43.17)	23.0
T4a	359 (70.67)	210 (71.19)	42.1	509 (39.21)	407 (40.02)	20.0
T4b	149 (29.33)	85 (28.81)	45.6	162 (12.48)	132 (12.98)	18.0
Lymph node metastasis (LNM)						
N0	–	–	–	31 (2.39)	18 (1.77)	31.0
N1	137 (26.97)	67 (22.71)	74.6	114 (8.78)	71 (6.98)	44.0
N2	172 (33.86)	100 (33.90)	40.8	341 (26.27)	232 (22.81)	31.0
N3a	161 (31.69)	96 (32.54)	44.4	518 (39.91)	424 (41.69)	18.0
N3b	38 (7.48)	32 (10.85)	14.1	294 (22.65)	272 (26.75)	16.0
Distant metastasis						
M0	481 (94.69)	271 (91.86)	46.3	1078 (83.05)	814 (80.04)	23.0
M1	27 (5.31)	24 (8.14)	20.8	220 (16.95)	203 (19.96)	15.0
Adjuvant treatment*						
None	195 (38.39)	115 (38.98)	42.9	–	–	–
Chemo	239 (47.05)	130 (44.07)	47.5	–	–	–
Chemo + Radio	72 (14.17)	48 (16.27)	41.9	–	–	–
Missing	2 (0.39)	2 (0.68)	–			
Staging system						
Sixth						
II	–	–	–	196 (15.10)	125 (12.29)	36.0
IIIa	204 (40.16)	110 (37.29)	54.3	435 (33.51)	310 (30.48)	26.0
IIIb	112 (22.05)	64 (21.69)	41.3	160 (12.33)	130 (12.78)	19.0
IV	192 (37.80)	121 (41.02)	37.0	507 (39.06)	452 (44.44)	17.0
Seventh						
IIIa	89 (17.52)	44 (14.92)	81.6	307 (23.65)	189 (18.58)	41.0
IIIb	158 (31.10)	85 (28.81)	45.3	436 (33.59)	335 (32.94)	22.0
IIIc	234 (46.06)	142 (48.14)	39.6	335 (25.81)	290 (28.52)	17.0
IV	27 (5.31)	24 (8.14)	20.8	220 (16.95)	203 (19.96)	15.0
Eighth						
IIIa	204 (40.16)	110 (37.29)	54.3	413 (31.82)	261 (25.66)	38.0
IIIb	205 (40.35)	110 (37.29)	48.7	417 (32.13)	325 (31.96)	21.0
IIIc	72 (14.17)	51 (17.29)	34.0	248 (19.11)	228 (22.42)	17.0
IV	27 (5.31)	24 (8.14)	20.8	220 (16.95)	203 (19.96)	15.0
Race						
African	–	–	–	219 (16.87)	175 (17.21)	22.0
Asian	508 (100.00)	295 (100.00)	44.4	309 (23.81)	239 (23.50)	21.0
Caucasian	–	–	–	769 (59.24)	603 (59.29)	21.0
Missing	–	–	–	1 (0.08)	0	–

Note

Chemo, chemotherapy; Radio, radiation therapy, MST, median survival time.

### 3.2 Construction and validation of the nomogram

Nine variables with prognostic capacity were evaluated using univariate analysis. Five variables, including age, tumor grade, tumor size, lymph nodes, and distant metastases were associated with OS significantly in the training set (Table 2). Multivariate Cox regression analysis confirmed that age (HR: 1.52, 95% CI: 1.19‐1.93), tumor size (for 5‐7 cm, HR: 1.35, 95% CI: 1.03‐1.75; for > 7 cm, HR :2.09, 95% CI: 1.48‐2.94), lymph nodes (for N2, HR: 1.37, 95% CI: 1.00‐1.87; for N3a, HR: 1.46, 95% CI: 1.06‐2.02; for N3b, HR: 3.43, 95% CI: 2.22‐5.29), and distant metastasis (HR: 1.69, 95% CI: 1.10‐2.59) remained independent prognostic predictors for OS (Table 2). The survival curves of nine risk variables are drawn in Figure S2A–i.

**TABLE 2:** Univariate and multivariate analysis of the training cohort

Characteristics	Univariate analysis		Multivariate analysis	
	HR (95% CI)	*P*‐value	HR (95% CI)	*P*‐value
Age (year）				
<63	1.00	–	1.00	–
≥63	1.43 (1.13‐1.81)	0.003	1.52 (1.19‐1.93)	<0.001
Gender				
Male	1.00	–	–	–
Female	0.90 (0.69‐1.19)	0.46	–	–
Location				
Upper third (C)	1.00	–	–	–
Middle third (M)	0.92 (0.70‐1.21)	0.55	–	–
Lower third (A)	1.13 (0.84‐1.53)	0.41	–	–
Missing	–	–	–	–
Tumor size (cm)				
<5	1.00	–	1.00	–
5‐7	1.48 (1.14‐1.92)	0.003	1.35 (1.03‐1.75)	0.03
>7	2.41 (1.72‐3.38)	<0.001	2.09 (1.48‐2.94)	<0.001
Tumor grade				
G1/G2	1.00	–	1.00	–
G3	1.35 (1.07‐1.70)	0.01	1.25 (0.99‐1.59)	0.06
T stage				
T4a	1.00	–	–	–
T4b	0.94 (0.73‐1.22)	0.66	–	–
Lymph node metastasis (LNM)				
N0				
N1	1.00	–	1.00	–
N2	1.43 (1.05‐1.95)	0.02	1.37 (1.00‐1.87)	0.05
N3a	1.54 (1.13‐2.11)	0.01	1.46 (1.06‐2.02)	0.02
N3b	3.74 (2.45‐5.73)	<0.001	3.43 (2.22‐5.29)	<0.001
Distant metastasis				
M0	1.00	–	1.00	–
M1	2.04 (1.35‐3.11)	<0.001	1.69 (1.10‐2.59)	0.02
Adjuvant treatment*				
None	1.00	–	–	–
Chemo	0.89 (0.69‐1.14)	0.36	–	–
Chemo + Radio	1.11 (0.79‐1.55)	0.56	–	–

Note

Chemo, chemotherapy; Radio, radiation therapy; HR, hazard ratio; CI, confidence interval.

**FIGURE 2:**
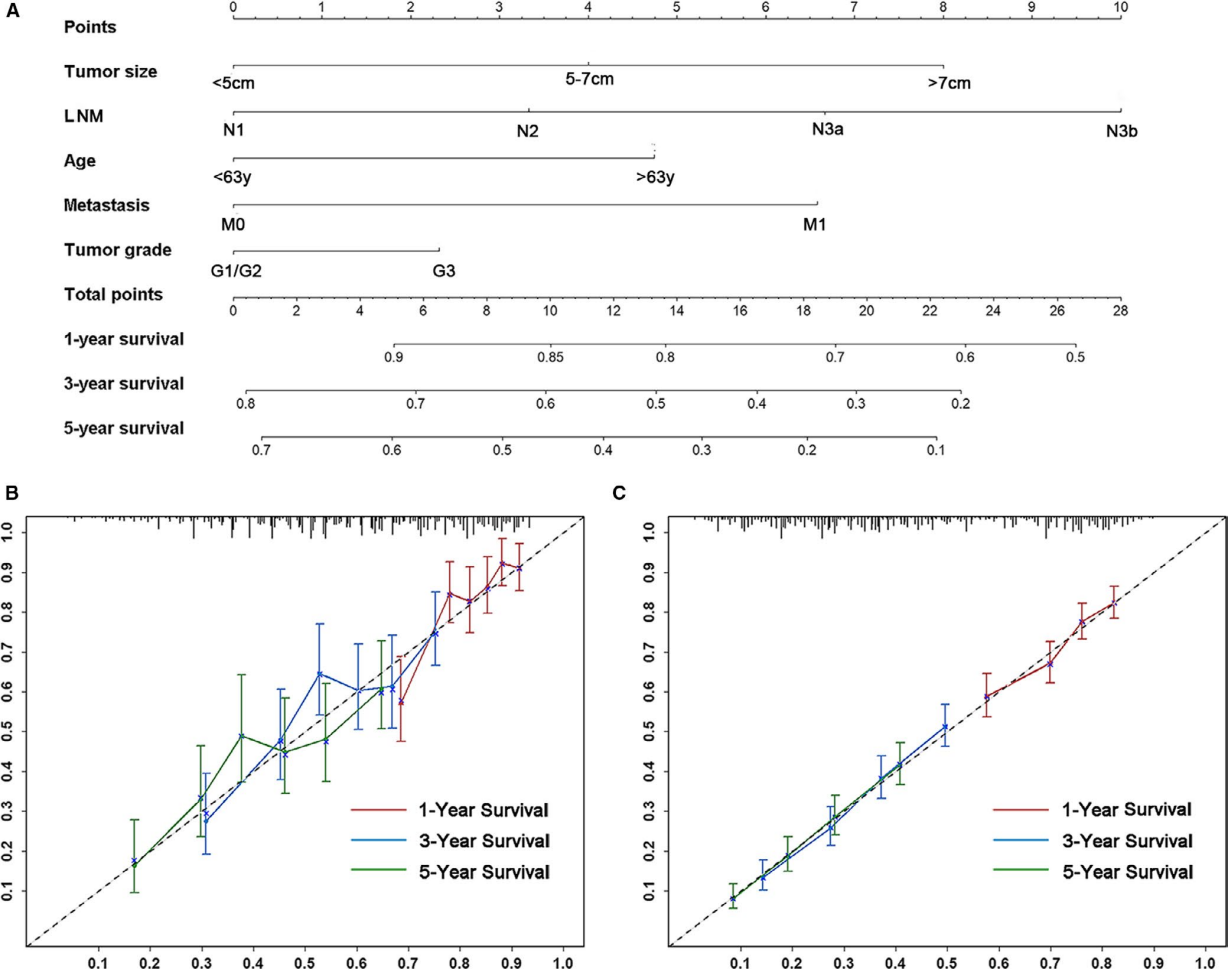
A, Nomogram conveys the results of prognostic models using clinicopathologic variables to predict 1‐, 3‐, and 5‐year overall survival rate of patients with stage III‐IV gastric cancer after racial resection. B, The calibration plot for nomogram in the internal validation. C, The calibration plot for nomogram in external set

Based on the minimum AIC, we constructed a nomogram to predict 5‐year OS for stage III‐IV GC patients following complete resection by incorporating five independent prognostic factors (Figure 2A). The resulting model was internally validated by the bootstrap method. The C‐index of the nomogram (0.645, 95% CI: 0.611‐0.679) was higher than that of the sixth TNM system (0.544, 95% CI: 0.512‐0.576, *P* < .05), the seventh TNM system (0.575, 95% CI: 0.543‐0.607, *P* < .05), and the eighth TNM system (0.568, 95% CI: 0.535‐0.601, *P* < .05) in the training cohort (Table 3). The calibration plot was depicted in Figure 2B, which appeared to show good consistency between the actual event and predicted event. The 1‐, 3‐, and 5‐ year AUC values of ROC were 0.697, 0.652, and 0.642, respectively, which were superior to the current TNM systems. (Figure S3A–C).

**TABLE 3:** Predictive validation for nomogram and AJCC staging systems

System	C‐index	95% CI	Z‐score
Training cohort			
AJCC sixth TNM	0.544	0.512‐0.576	2.64
AJCC seventh TNM	0.575	0.543‐0.607	4.50
AJCC eighth TNM	0.568	0.535‐0.601	4.01
Nomogram	0.645	0.611‐0.679	8.32
Validation cohort			
AJCC sixth TNM	0.592	0.574‐0.610	10.52
AJCC seventh TNM	0.609	0.591‐0.627	12.01
AJCC eighth TNM	0.611	0.593‐0.629	12.40
Nomogram	0.626	0.612‐0.640	13.90
Race			
Asian	0.644	0.609‐0.679	8.01
African	0.604	0.561‐0.647	4.69
Caucasian	0.628	0.604‐0.652	10.67

Note

AJCC, American Joint Committee on Cancer; C‐index, Harrell's concordance‐index; CI, confidence interval.

In the external validation set, the nomogram showed a C‐index of 0.626 (95% CI: 0.612‐0.640) for the evaluations of death risk, which was higher than that of the eighth (0.611, 95% CI: 0.593‐0.629, *P *< .05), seventh (0.609, 95% CI: 0.591‐0.627, *P *< .05), and sixth (0.592, 95% CI: 0.574‐0.610, *P *< .05) AJCC TNM systems (Table 3). The calibration plots displayed an excellent agreement in the external validation cohort for 1‐, 3‐, and 5‐ year OS (Figure 2C). The AUC for nomogram were higher than for the AJCC staging systems which indicated the good discriminative ability of the nomogram (Figure S3D–F).

We further divided the validation cohort into three subgroups according to ethnicity (Asian, African, and Caucasian). The C‐index of this nomogram was the highest in Asian population (C‐index: 0.644, 95% CI, 0.609‐0.679) among all ethnic subgroups (African, 0.604, 95% CI, 0.561‐0.647; Caucasians, 0.628, 95% CI: 0.604‐0.652) (Table 3). Similarly, the AUC values at 5 years OS for nomogram was higher in Asians than in Africans and Caucasians (Figure S3H–i). The calibration plots for the nomogram in three subgroups showed accurate predictive ability (Figure S4A–C). These findings suggested that our nomogram might be more useful in the Asian population.

### 3.3 Performance of the nomogram in stratifying risk of patients

**FIGURE 3:**
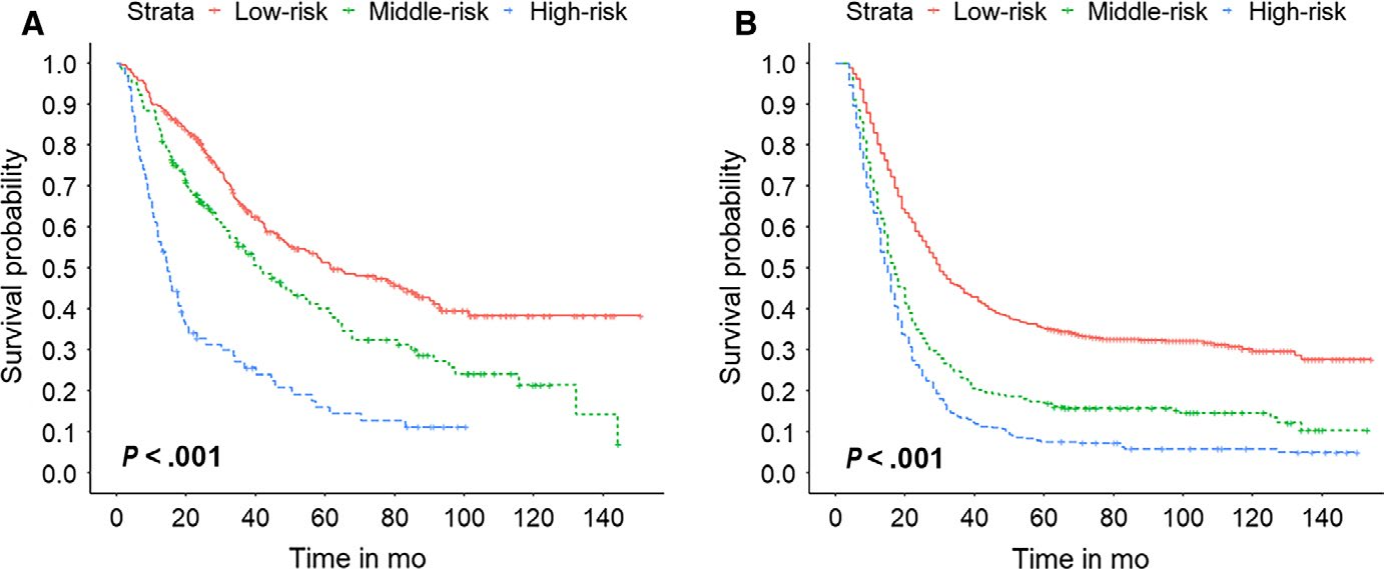
Survival curves stratified by the risk score calculated by the total points of the nomogram (low‐risk, <16.07; middle‐risk, 16.07‐19.40). A, Survival curves for different risk groups in the internal validation cohort. B, Survival curves for different risk groups in the external validation cohort

We calculated the total points associated with OS based on the nomogram in the internal validation set and external validation set. Total points as a continuous variable with normal distribution were shown in Figure S5. The optimal cutoff value of total points was selected to be 16.07 and 19.40 by the X‐tile analysis. Patients were divided into three subgroups (high‐risk, >19.40; middle‐risk, 16.07‐19.40; low‐risk, <16.07). The MST of the high‐, middle‐, and low‐risk groups was 14.7, 41.3, and 61.5 months in internal validation set, respectively. Also, that in external validation cohort was 15.0, 17.0, and 30.0 months, respectively. The OS probability for the three subgroups in the training set were 50.7%, 37.4%, and 17.6% (*P *< .001, Figure 3A), respectively. Similarly, we observed significant differences in the external validation set (OS: low‐risk, 31.8%, middle‐risk, 14.3%, and high‐risk, 6.0%; *P* < .001, Figure 3B). This stratification could effectively discriminate the survival outcomes for the three proposed subgroups in both training and validation sets.

## 4 DISCUSSION

In this study, we analyzed 1806 GC patients at stage III‐IV who underwent racial resection. According to five independent prognostic factors (age, tumor size, tumor grade, N stage, and distant metastasis), we constructed a novel nomogram to predict the outcomes of III‐IV GC after surgical treatment. This nomogram showed higher prognostic efficacy and good performance in internal and external cohorts than the AJCC staging systems. It will be helpful to identify the independent factors of survival in GC patients at stage III‐IV and to guide the choice of individualized treatment.

Fewer than 28% of patients with advanced or metastases disease survive 5 years after surgical treatment because of multicentric tumors, vascular invasion, and distant metastases.^23^ Combining surgery and radiotherapy could improve OS.^24^ However, treatment (chemotherapy and radiotherapy) was not an independent risk factor for advanced GC in this study. The finding was similar to results of another study.^25^

The Japanese treatment guidelines recommend adjuvant therapy combined with surgery for advanced GC.^26^ The MST of GC patients with stage IV is 13‐16 months.^27^, ^28^, ^29^ In our study, the MST of GC patients at stage IV who underwent surgical resection was 20.8 months. Considering chemotherapy resistance and cumulative adverse events, surgery was recommended as a part of a comprehensive treatment strategy at some time point during the entire treatment course. Combined resection of the metastatic site (which is performed at the surgeon's discretion based on the laparotomy findings) is mainly responsible for the high risk of death.^30^, ^31^ However, current TNM staging systems in predicting OS of cancer have limitations. Thus, it is necessary to identify patients at the high‐risk level after racial resection.

Five independent prognostic factors were integrated to construct the novel nomogram. Multiple studies have revealed that patients diagnosed at the age of < 50 and > 80 years had higher risks of death.^8^, ^32^ Tumor differentiation grade is an independent prognostic factors of survival in GC patients.^7^, ^33^ Tumor size ≥ 4.8 cm in diameter represents poor prognosis for GC pathologic grade.^7^, ^34^, ^35^ We also found that the differences in hazard risk (HR) among the three subgroups (<5.0 cm, 5.0‐7.0 cm, and > 7.0 cm) were significant (*P *< .05).

The 5‐year survival probability of patients with GC in the Asian cohort was higher than that of the African and Caucasian cohorts (5‐year OS 26.21%, 23.74%, and 24.16%, respectively, *P *< .001) in our external cohorts, and the results was supported by a previous study.^36^ Meanwhile, our nomogram exhibits a better performance in the Asian population. These differences may due to genetic inheritance, culture, and dietary habits of the various ethnicities.

There are some limitations to this study. First, a large multicenter survey is needed in the future to verify the conclusions of this investigation, because the study only collected 508 patients to construct a nomogram. Second, the C‐index of the nomogram was 0.626 in external cohort, which was smaller than that of the internal cohort (C‐index: 0.645). The explanation may be that the ethnic composition ratio was different between internal and external sets. Third, in the training set, we found that the predictive ability of the seventh TNM classification was higher than that of the eighth AJCC. We did not observe the same regularity in the validation cohort. The explanation may be that patients were captured in the training cohorts without examining more than 16 lymph nodes. Finally, there was reporting bias as to gastroesophageal junction tumors because the study was based on a multi‐institutional “gastric” database and clinical statistics of patients were collected by report ten years prior when esophagogastric junction cancer was not clearly defined.

## 5 CONCLUSION

In summary, we constructed and validated an accurate prognostic nomogram model for GC patients at stage III‐IV based on age, tumor grade, tumor size, lymph nodes, and distant metastases clinical variables. The nomogram showed powerful predictive ability by internal and external validation, which is more accurate and useful than the current AJCC staging systems. This tool might help clinicians conduct personalized prognostic evaluations and could be applied as a widely applied tool for future clinical evaluation.

## DATA SHARING STATEMENT

The datasets used or analyzed during the current study are available from the corresponding author on reasonable request.

## COMPETING INTEREST

All authors had declared that they have no interest conflicts.

## ETHICAL DISCLOSURE

The authors declared that they have followed the principles in the Declaration of Helsinki for all human or animal experimental investigations. Informed consent has been obtained from the participants involved for studies involving human subjects.

## AUTHOR CONTRIBUTIONS

Conceptualization: Zhiying Gao and Jing Ni; investigation:, Zhiying Gao, Hui Ding, Caiwang Yan, Gang Li, and Chuanli Ren; writing–manuscript: Zhiying Gao and Jing Ni; writing—review and editing: Feng Pan and Guangfu Jin; funding acquisition: Guangfu Jin;

## Supporting information

Fig S1

Fig S2

Fig S3

Fig S4

Fig S5

Supplementary Material
