# Supplementary figures and images for: A nomogram for prediction of stage III/IV gastric cancer outcome after surgery: A multicenter population‐based study
Source: Cancer Med. 2020 Jun 15;9(15):5490–9. doi: 10.1002/cam4.3215 (PMC7402842; doi:10.1002/cam4.3215)

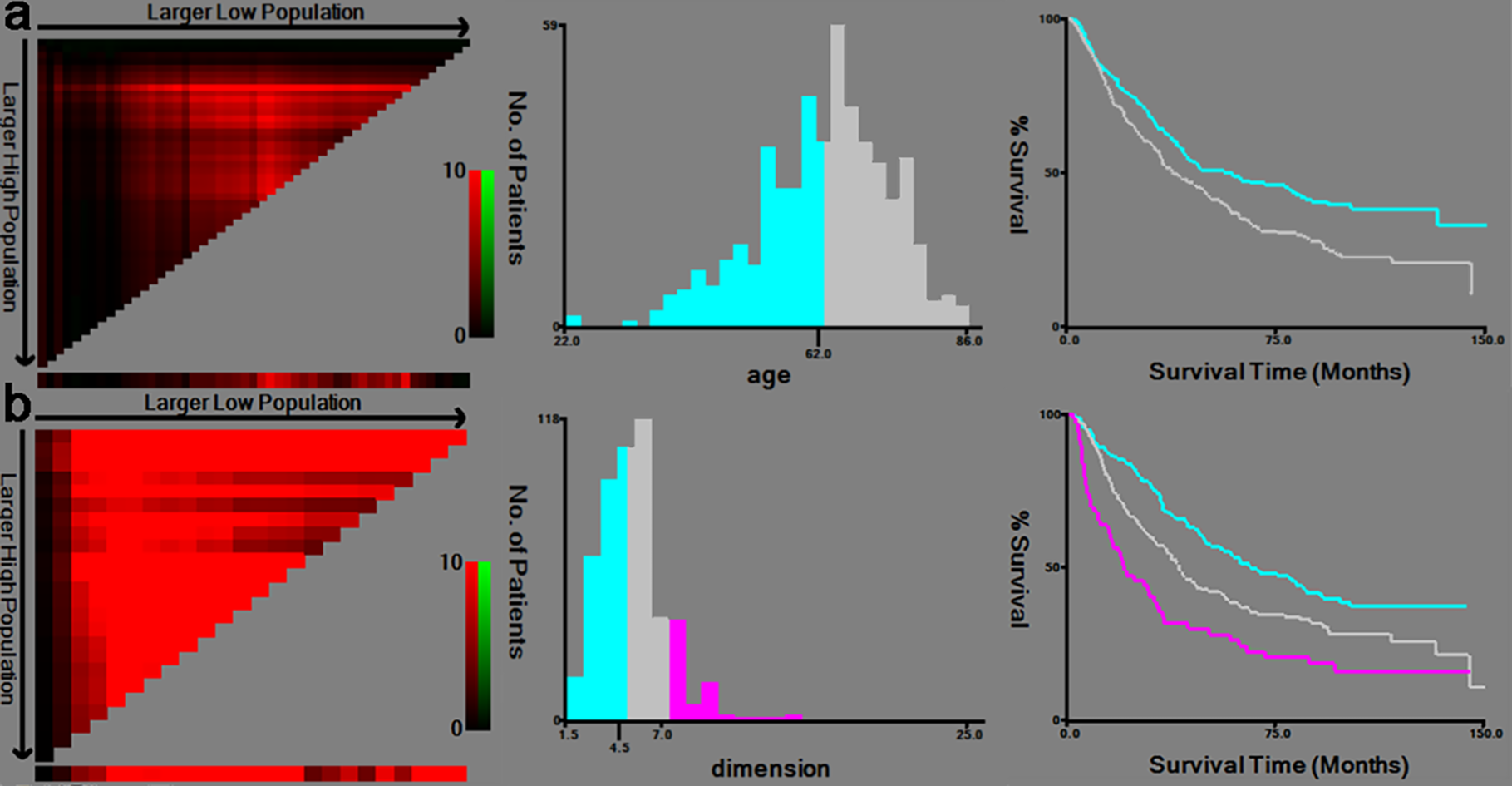

Supplement: Supplementary file 1 — Fig S1 [file CAM4-9-5490-s001.tif]

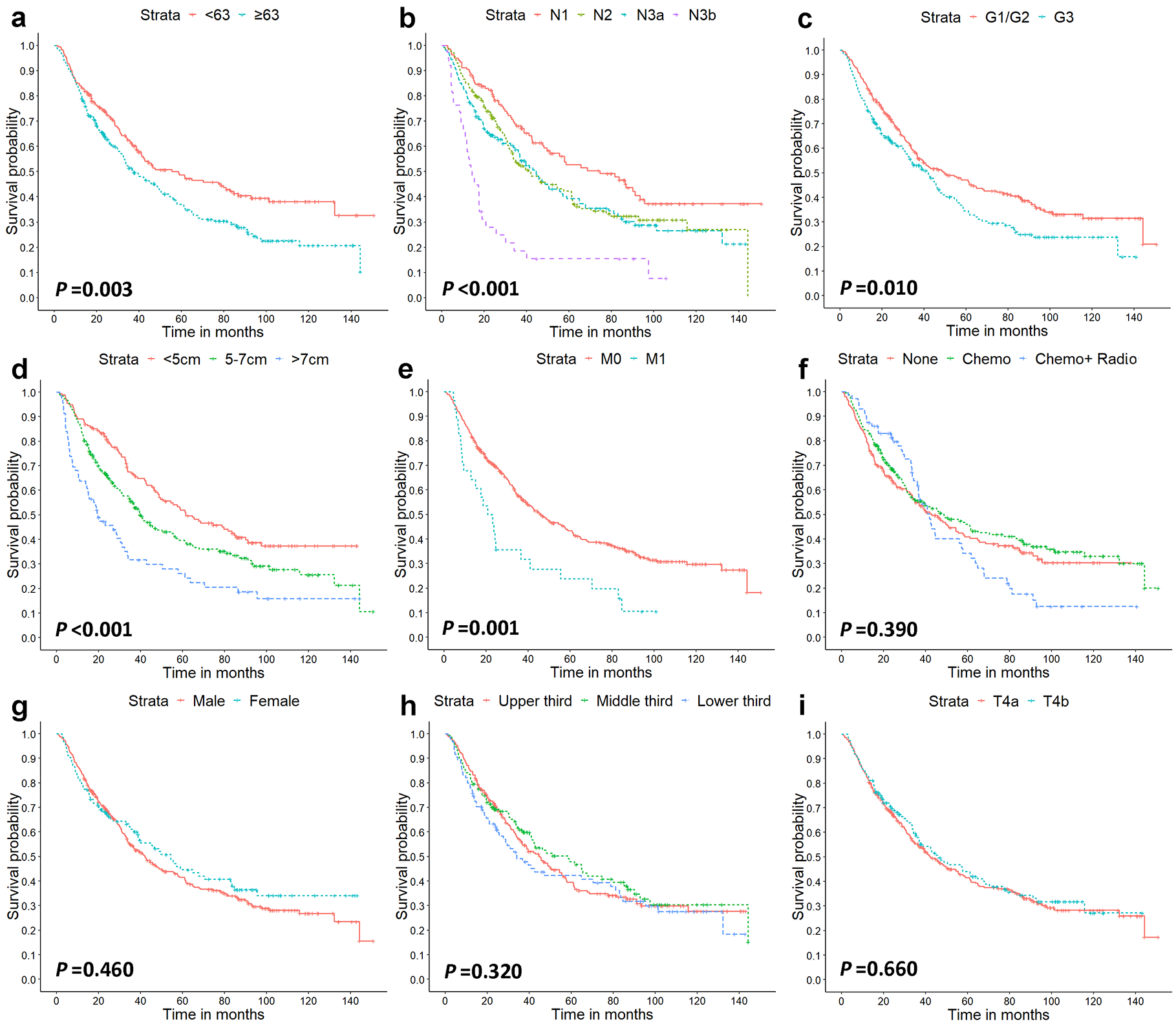

Supplement: Supplementary file 2 — Fig S2 [file CAM4-9-5490-s002.tif]

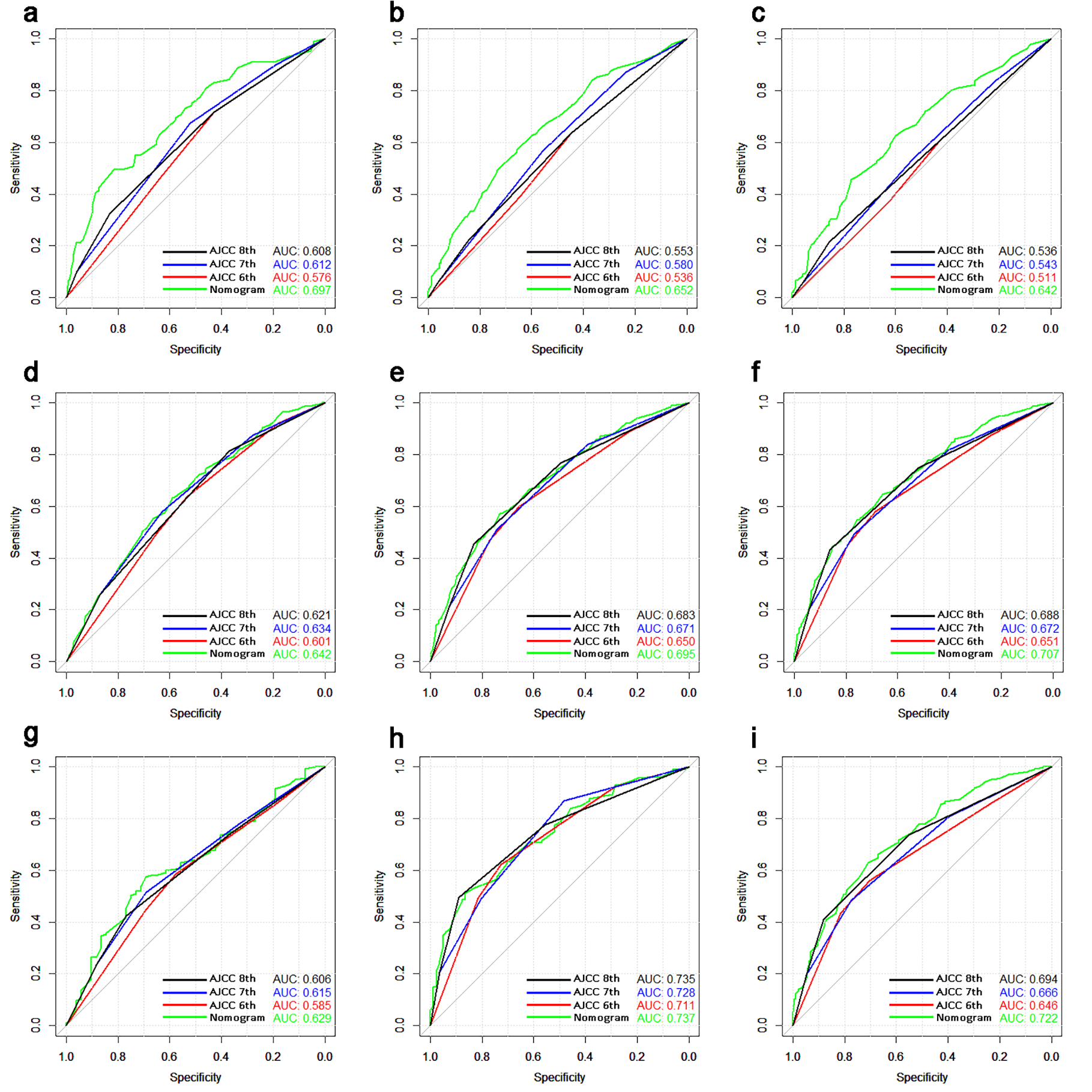

Supplement: Supplementary file 3 — Fig S3 [file CAM4-9-5490-s003.tif]

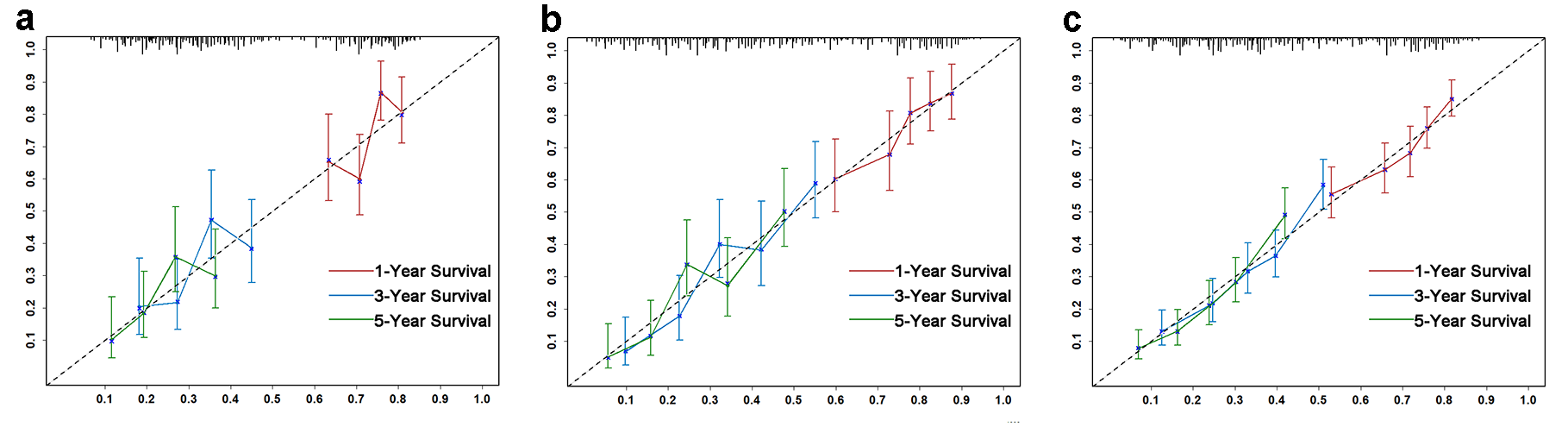

Supplement: Supplementary file 4 — Fig S4 [file CAM4-9-5490-s004.tif]

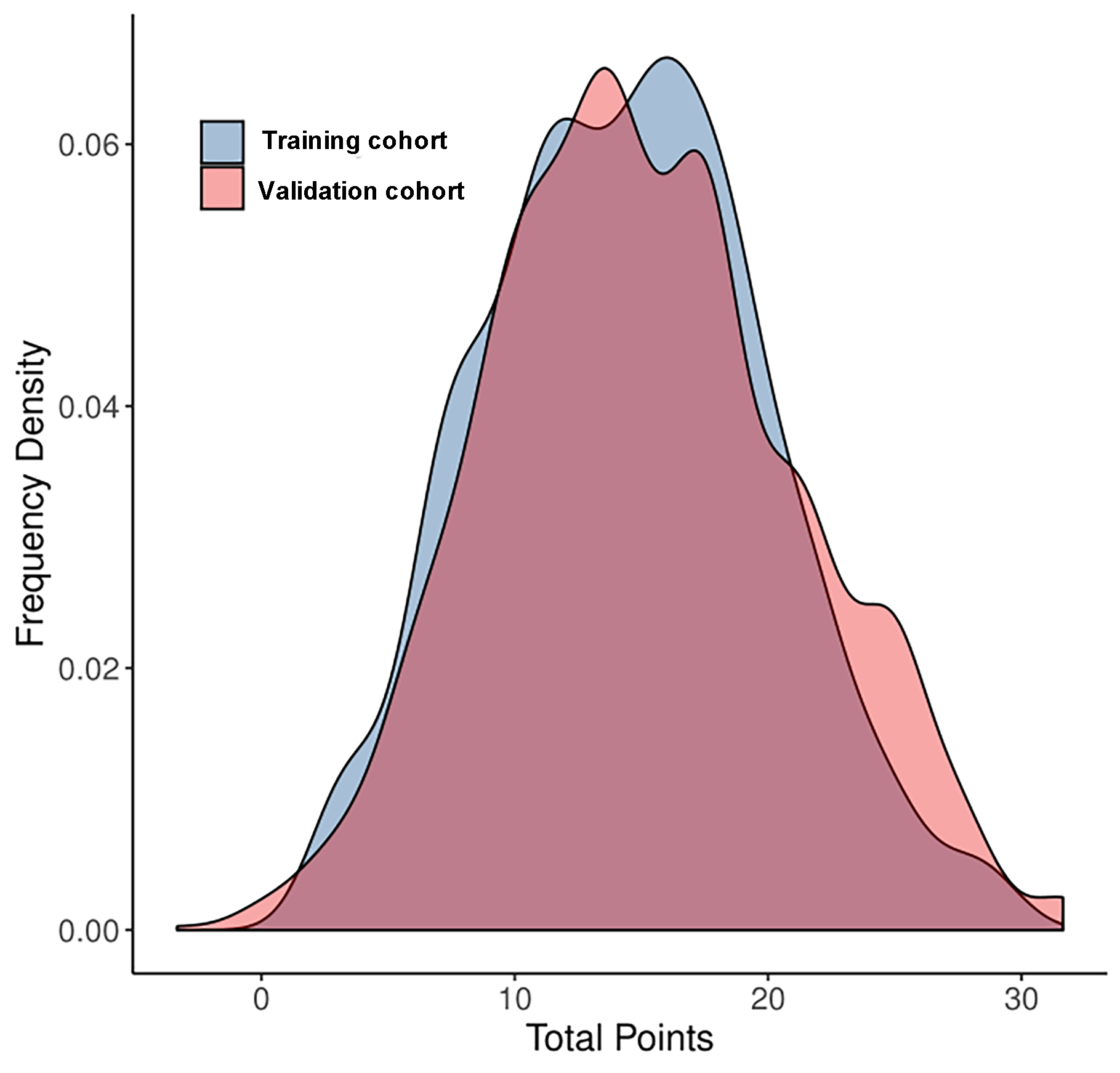

Supplement: Supplementary file 5 — Fig S5 [file CAM4-9-5490-s005.tif]
