# Supplementary material for: A nomogram for prediction of stage III/IV gastric cancer outcome after surgery: A multicenter population‐based study
Source: Cancer Med. 2020 Jun 15;9(15):5490–9. doi: 10.1002/cam4.3215 (PMC7402842; doi:10.1002/cam4.3215)
Supplement: Supplementary file 6 — Supplementary Material [file CAM4-9-5490-s006.docx]

**Supplementary Figures**


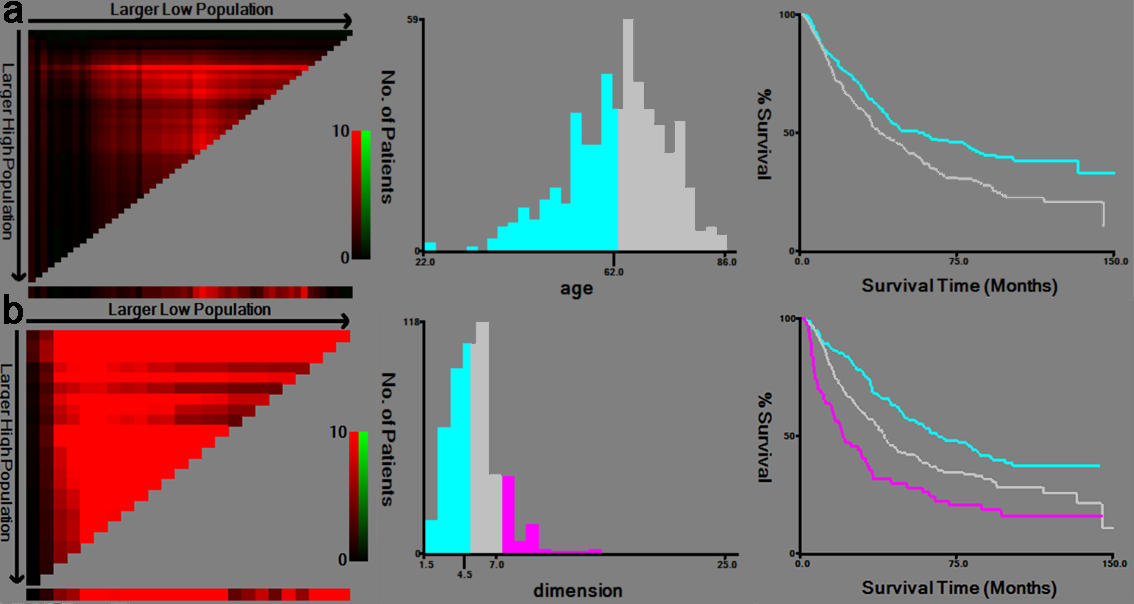


**Supplementary Figure 1.** X-tile analyses was performed to determine the optimal cutoff values for age and tumor size. The optimal cutoff values highlighted by the black circles in left panels are shown in histograms of the entire cohort (middle panels), and Kaplan-Meier plots are displayed in right panels. *P*-values were determined by using the cutoff values defined in training sets and applying them to validation sets. (a) The optimal cutoff value of age was 63 years old (*P*<0.001). (b) The cutoff of tumor size was 5.0 cm and 7.0 cm (*P*<0.001).


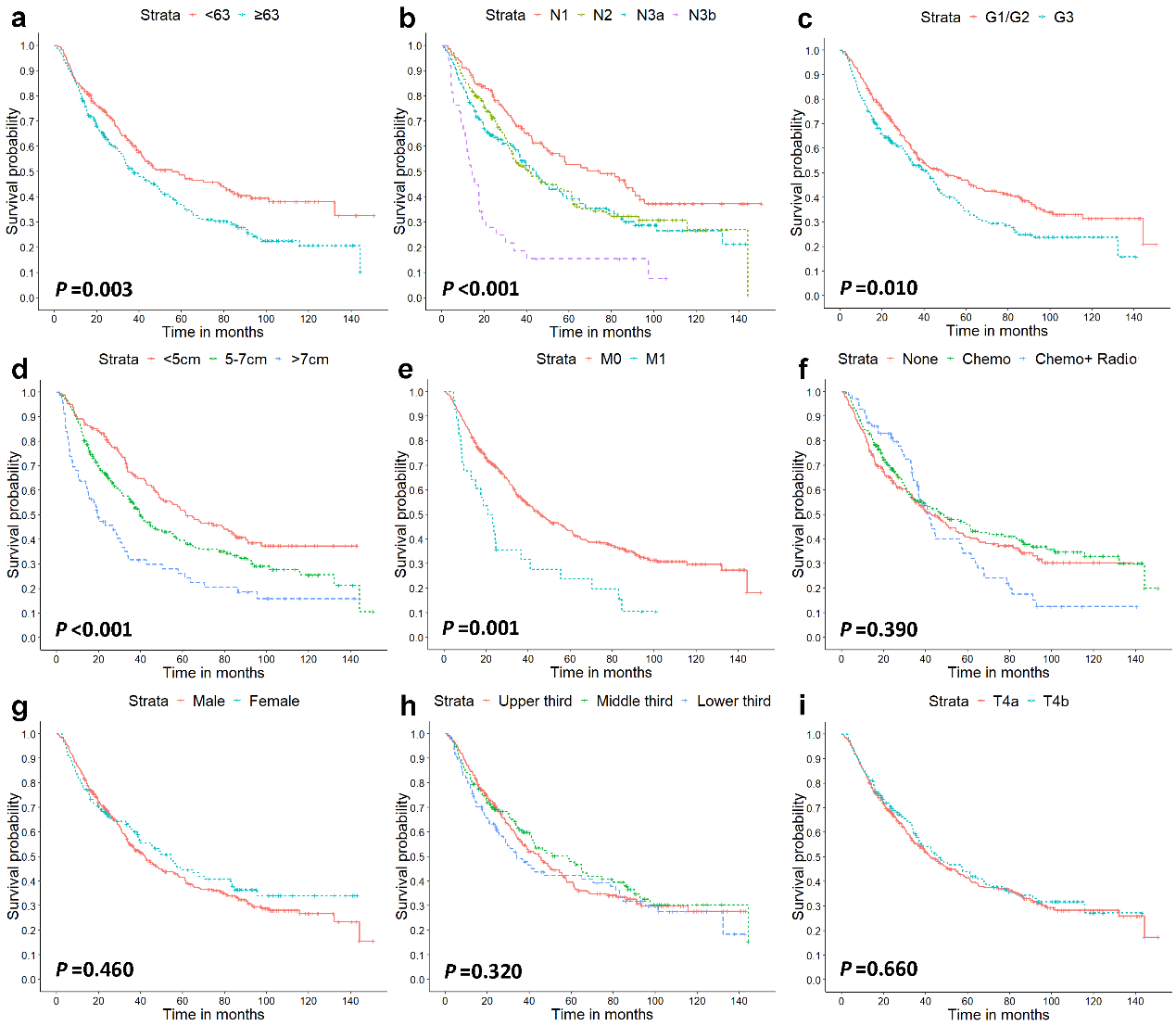


**Supplementary Figure 2.** Kaplan -Meier analysis was performed to evaluate the survival of patients divided by prognostic factors in the training cohort. (a) Survival curve for age (≥63 years old). (b) Survival curve for numbers of lymph node metastasis (N1, N2, N3a, N3b). (c) Survival curve for tumor grade (G1/G2, G3). (d) Survival curve for tumor size (< 5cm, 5-7cm, >7cm). (e) Survival curve for distant metastasis (M0, M1). (f) Survival curve for treatment (none, only chemotherapy, chemotherapy and radiotherapy). (g) Survival curve for sex (Male, Female). (h) Survival curve for tumor site (Upper third, Middle third, Lower third). (i) Survival curve for numbers of the depth of tumor invasion (T stage) (T4a, T4b).


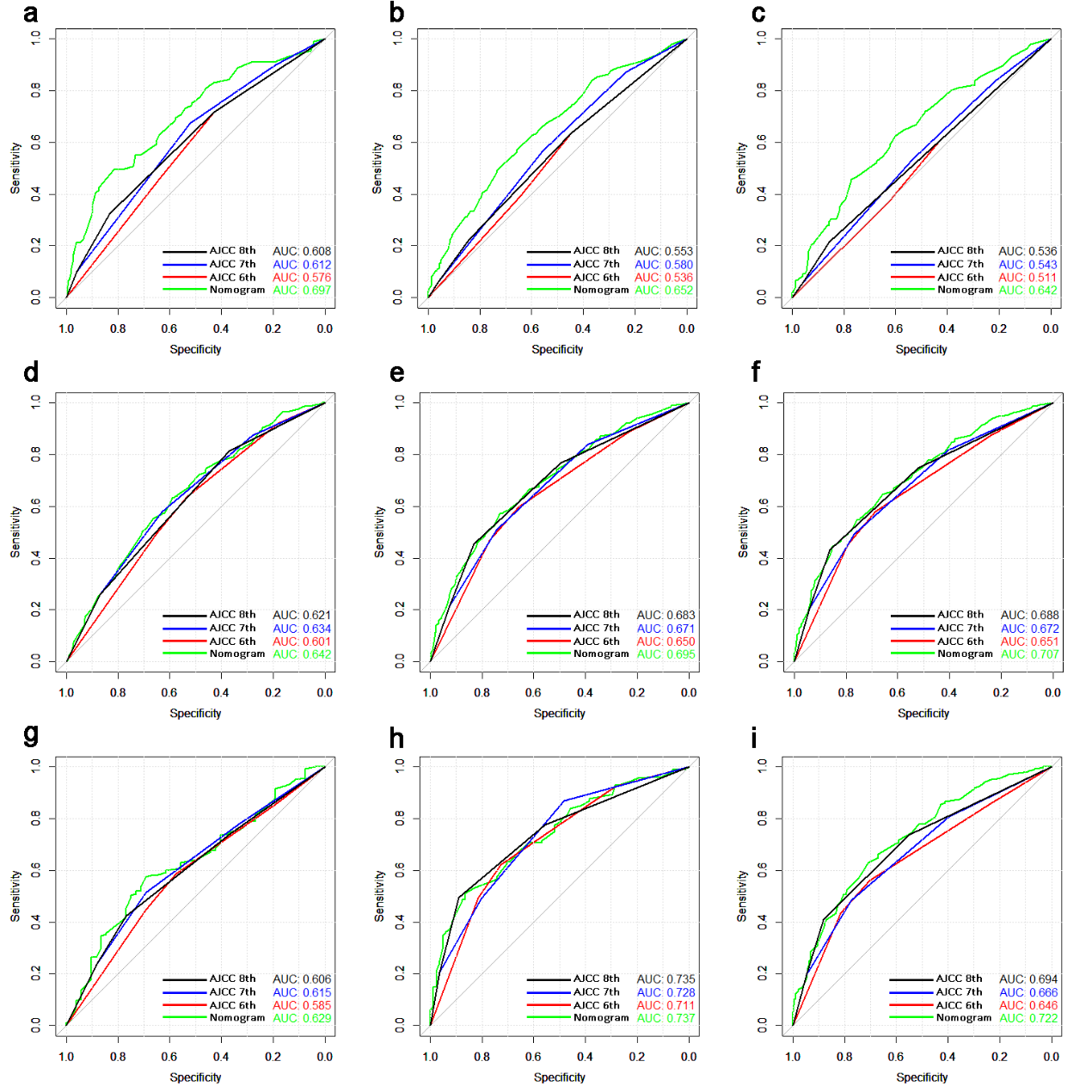


**Supplementary Figure 3.** The receiver operating characteristic curve (ROC) and area under curve (AUC) were used to measure model’s ability to distinguish events and no events. (a) (b) (c): ROC for predicting the rate of 1-, 3-, and 5-year overall survival (OS) respectively in training cohort. (d) (e) (f): ROC for the rate of 1-,3- and 5-year OS rate respectively in external validation cohort. (g) (h) (i): ROC for the rate of 5-year OS in African, Asian, and Caucasian population respectively.

**
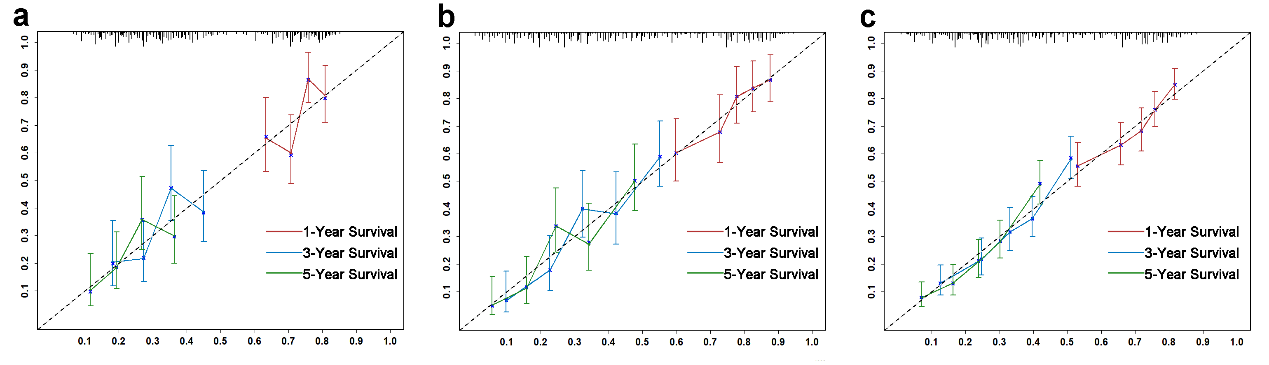
**

**Supplementary Figure 4.** (a) Calibration plot for nomogram in the African population. (b) Calibration plot for nomogram in the Asian population. (c) Calibration plot for nomogram in the Caucasian population.


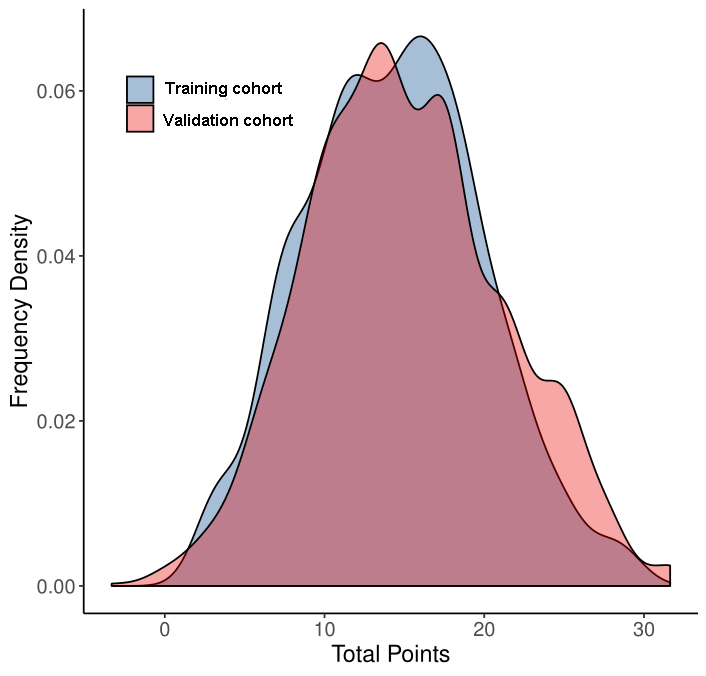


**Supplementary Figure 5.** Frequency histogram for total points calculated based on nomogram in the training and validation cohort.
